# ICTV Virus Taxonomy Profile: Hadakaviridae 2023

**DOI:** 10.1099/jgv.0.001820

**Published:** 2023-01-05

**Authors:** Yukiyo Sato, Massimo Turina, Sotaro Chiba, Ryo Okada, Muhammad F. Bhatti, Ioly Kotta-Loizou, Robert H. A. Coutts, Hideki Kondo, Sead Sabanadzovic, Nobuhiro Suzuki, ICTV Report Consortium

**Affiliations:** 1 Institute of Plant Science and Resources, Okayama University, Kurashiki 710-0046, Japan; 2 Institute for Sustainable Plant Protection-CNR, Torino 10135, Italy; 3 Graduate School of Bioagricultural Sciences, Nagoya University, Nagoya 464-0861, Japan; 4 Horticultural Research Institute, Ibaraki Agricultural Center, Kasama 319-0292, Japan; 5 Atta-ur-Rahman School of Applied Biosciences, National University of Sciences and Technology, Sector H-12, 44000 Islamabad, Pakistan; 6 Department of Life Sciences, Faculty of Natural Sciences, Imperial College London, London SW7 2AZ, UK; 7 Department of Clinical, Pharmaceutical and Biological Sciences, School of Life and Medical Sciences, University of Hertfordshire, Hatfield AL10 9AB, UK; 8 Department of Biochemistry, Molecular Biology, Entomology and Plant Pathology, Mississippi State University, Mississippi State, MS 39762, USA

**Keywords:** *Hadakaviridae*, ICTV Report, taxonomy

## Abstract

The family *Hadakaviridae*, including the genus *Hadakavirus*, accommodates capsidless viruses with a 10- or 11-segmented positive-sense (+) RNA genome. Currently known hosts are ascomycetous filamentous fungi. Although phylogenetically related to polymycovirids with a segmented double-stranded RNA genome and certain encapsidated picorna-like viruses, hadakavirids are distinct in their lack of a capsid (‘hadaka’ means naked in Japanese) and their consequent inability to be pelleted by conventional ultracentrifugation; they show ribonuclease susceptibility in host tissue homogenates. This is a summary of the International Committee on Taxonomy of Viruses (ICTV) Report on the family *Hadakaviridae,* which is available at ictv.global/report/hadakaviridae.

## Virion

Hadakavirids (members of the family *Hadakaviridae*) have no true virion (Table 1). Accordingly, hadakavirids cannot be pelleted by ultracentrifugation [1] and the hadakavirid positive-sense (+) RNA genome and replicative form double-stranded (ds) RNA are accessible by and susceptible to exogenously added ribonuclease in host crude tissue homogenate under conditions in which encapsidated viral RNA remains undigested [1,2]. Mechanisms of hadakavirid genomic RNA protection in the host are yet to be elucidated.

**Table 1.** Characteristics of members of the family *Hadakaviridae*

Example:	hadaka virus 1 (LC519840–LC519850), species *Hadakavirus nanga*, genus *Hadakavirus*
Virion	No known virions (capsidless)
Genome	Multi-segmented (10 or 11) linear, positive-sense (+) RNAs comprising 14–15 kb in total; segments are 0.9–2.5 kb
Replication	Double-stranded replicative forms accumulate abundantly in infected fungal hosts. The cellular replication site remains unknown.
Translation	From non-polyadenylated monocistronic genomic RNAs
Host range	Fungi
Taxonomy	Realm *Riboviria*; kingdom *Orthornavirae*, phylum *Pisuviricota*; the family includes the genus *Hadakavirus* and the species *Hadakavirus nanga*

## Genome

**Fig. 1.**
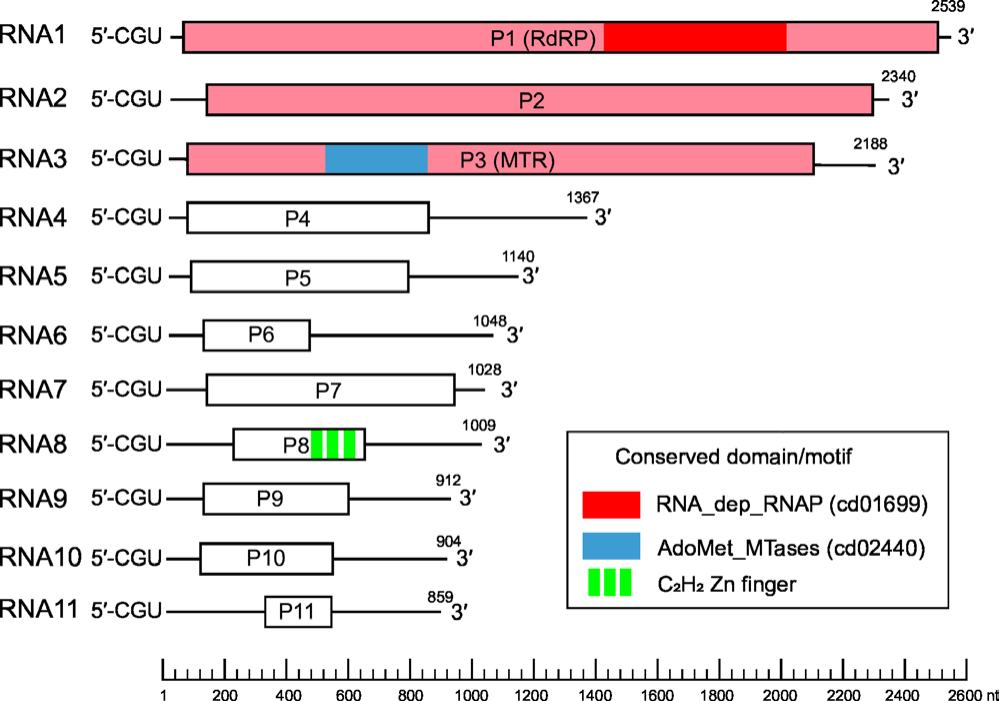
Genome organization of hadaka virus 1 isolate 7 n (LC519840–50). Open reading frames are indicated by boxes. The three genes homologous between hadakavirids and polymycovirids are coloured pink: P1 (RdRP, RNA-dependent RNA polymerase), P2 (unknown function) and P3 (MTR, methyltransferase). Other coloured regions indicate conserved domains or motifs identified previously [1]

Hadakavirids have a genome comprising 10- or 11 monocistronic (+) RNA segements (Fig. 1). The 5′-terminal three nucleotides (CGU) are conserved in all segments. The largest three genomic segments (RNA1–RNA3) encode proteins homologous to those of members of the family *Polymycoviridae*, namely an RNA-dependent RNA polymerase (RdRP), a hypothetical protein of unknown function and a methyltransferase [1,3]. While the genome of hadaka virus 1 isolate 7 n includes RNA8 that encodes a hypothetical protein containing a C_2_H_2_-type zinc finger motif [1], this genomic segment is missing in hadaka virus 1 isolate 1 NL [2]. The functions of virus proteins encoded by segments 2 and 4–11 are unknown.

## Replication

The replication mechanism has not been investigated.

## Pathogenicity

No pathogenic effect on the host has been reported for isolates of hadaka virus 1 [1,2]. However, a related, unclassified virus, Colletotrichum fructicola RNA virus 1, causes mild growth inhibition in a phytopathogenic fungus [4].

## Taxonomy

Current taxonomy: ictv.global/taxonomy. Hadakavirids are most closely related to members of the family *Polymycoviridae* [1,3, 5]. In turn, the RdRPs of hadadakavirids and polymycovirids show phylogenetic affinity to those of (+) RNA viruses in the phylum *Pisuviricota* (extended ‘picornavirus supergroup’), such as the members in the families *Astroviridae* and *Caliciviridae*. Capsidless hadakavirids are regarded as (+) RNA viruses, based largely on their phylogenetic affinity of their RdRP. This taxonomic placement of hadakavirids is reminiscent of that of other capsidless RNA viruses, e.g. members of the family *Hypoviridae* [6]. The catalytic core residues of hadaka/polymyco RdRPs are ‘GDNQ’, which is found in some negative-sense mononegaviruses, rather than ‘GDD’, characteristic of most (+) RNA and dsRNA viruses.

## Resources

Full ICTV Report on the family *Hadakaviridae*: ictv.global/report/hadakaviridae.

## Abbreviations

(+): positive-sense
ds: double-stranded
RdRP: RNA-dependent RNA polymerase
